# Study of Generalized Phase Spectrum Time Delay Estimation Method for Source Positioning in Small Room Acoustic Environment

**DOI:** 10.3390/s22030965

**Published:** 2022-01-26

**Authors:** Vladimir Faerman, Valeriy Avramchuk, Kirill Voevodin, Ivan Sidorov, Evgeny Kostyuchenko

**Affiliations:** 1 Laboratory for Acquisition, Processing and Manipulating Biological Signals, Institute of System Integration and Security, Tomsk State University of Control Systems and Radioelectronics, 40 Lenina Ave., 634050 Tomsk, Russia; key@fb.tusur.ru; 2 Department of Complex Information Security of Computer Systems, Faculty of Security, Tomsk State University of Control Systems and Radioelectronics, 40 Lenina Ave., 634050 Tomsk, Russia; avs@fb.tusur.ru (V.A.); Kirill.Voevodin2@infotecs.ru (K.V.); 3 Irkutsk Supercomputer Center of SB RAS, 134, Lermontova, 664033 Irkutsk, Russia; ivan.sidorov@icc.ru

**Keywords:** generalized phase spectrum, time delay estimation, indoor positioning, room acoustics, sensors array

## Abstract

This paper considers the application of signal processing methods to passive indoor positioning with acoustics microphones. The key aspect of this problem is time-delay estimation (TDE) that is used to get the time difference of arrival of the source’s signal between the pair of distributed microphones. This paper studies the approach based on generalized phase spectrum (GPS) TDE methods. These methods use frequency-domain information about the received signals that make them different from widely applied generalized cross-correlation (GCC) methods. Despite the more challenging implementation, GPS TDE methods can be less demanding on computational resources and memory than conventional GCC ones. We propose an algorithmic implementation of a GPS estimator and study the various frequency weighting options in applications to TDE in a small room acoustic environment. The study shows that the GPS method is a reliable option for small acoustically dead rooms and could be effectively applied in presence of moderate in-band noises. However, GPS estimators are far less efficient in less acoustically dead environments, where other TDE options should be considered. The distinguishing feature of the proposed solution is the ability to get the time delay using a limited number of the adjusted bins. The solution could be useful for passively locating moving emitters of narrow-band continual noises using computationally simple frequency detection algorithms.

## 1. Introduction

The problem of time-delay estimation (TDE) is to measure the difference in the time of arrival of signals recorded by space-separated sensors. This task is relevant for many applications, including those which are related to signal source localization [1]. The position of the object can be determined on the straight line [2,3], on the plane [4,5], and in space [6,7,8] depending on the location and the number of sensors.

The use of TDE methods is typical for those areas of technology where there is a need for the passive location of objects emitting signals. The physical nature of the signal, however, is not essential. Among practical applications, we can highlight the pipeline leaks position determination [2,3], local mobile objects positioning [9], passive radio positioning [1], etc. In recent years, the problem of TDE has become more relevant in connection with the spread, on the Internet, of concepts and services providing contactless control of household appliances [10], automatic tracking of objects [7], as well as in the sensor systems of robotic devices [11]. A common problem in the implementation of each of the listed services is the need for signal sources spatial discrimination, which normally requires TDE. Also, it should be noted that the development of industrial Internet applications requires solving the TDE problem for the time synchronization of data coming from asynchronous and spatially distributed sensors [11].

TDE methods and algorithms form a broad subject area. At present different approaches for TDE are known. A number of reviews have been devoted to the classification and systematization of TDE algorithms for numerous and diverse applications, in particular [8,12,13,14,15]. This paper compares well-known but seldom used TDE algorithms based on estimating the phase shift (GPS TDE) between signals.

Even though the frequency-domain TDE technique was originally proposed by Piersol [16] and developed by Zhen and Zi-Quang [15] back in the 1980s, studies devoted to its applications are relatively rare. This could be because the practical implementation of the GPS TDE technique is not as straightforward as the implementation of GCC TDE. Efficient implementation requires unwrapped phase spectrum estimation and time lag extraction which can be performed in various ways. This applies some limitations on using well-described GPS TDE algorithms [14] for different practical tasks. With this paper, we will propose an implementation applicable for most typical TDE applications, such as pipeline leak locating [2] or acoustic intrusion detection [4].

Related studies considering TDE for sound source positioning in room acoustic environment have been carried out before, for instance, in [7,8]. However, GPS TDE or similar frequency-domain techniques were not considered there. Variations of CPS TDE are compared in [14] in the different applications of locating the acoustic source, but the single path propagation model was used to simulate a practical case. The single path propagation model is considered not accurate [7,8] for a small room reverberation environment, so the conclusions of [14] could not be extrapolated to this application without further research. In [17], a hardware implementation of an indoor positioning system based on the phase correlation TDE algorithm was proposed, however, only substitutional research was carried out within the framework of the signal processing.

## 2. Materials and Methods

The most studied and widespread TDE technique is based on cross-correlation functions computation (CCF) [2]. CCFs are calculated for different time series pairs of sampled microphone signals, based on the position of the maximum in a correlogram. An alternative to the TDE correlation methods are phase-frequency methods, suggested firstly in [17]. Unlike correlation methods which analyze signals in the time domain, phase methods operate with signals frequency-domain representations. This section is devoted to the phase methods of TDE.

**Figure 1:**
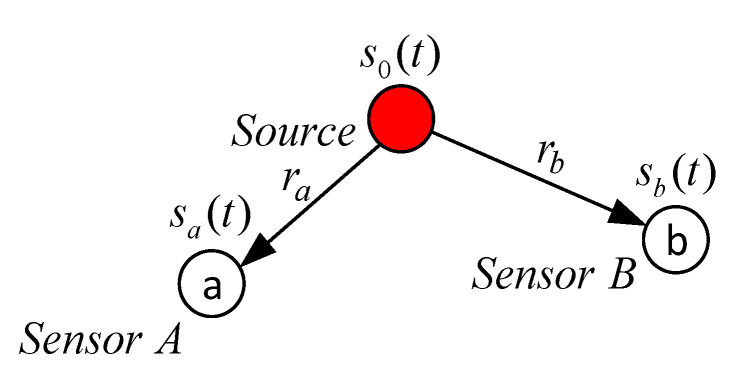
TDE with two sensors.

This paper considers the simplest case with two sensors, shown in Figure 1. Obviously, two sensors are not enough for unambiguous signal source localization on a plane or in space [11]. Depending on the relative sensor’s position and the position of the signal source, a pair of microphones may be sufficient to determine the direction towards the object. In general cases, at least three sensors are required to determine the position of the source in a room [16]. In this case, the signals of the sensors array can be processed both simultaneously and in pairs [8]. The latter means that the algorithm considered in the paper can be used to localize the signal source in a room using three or more microphones.

### 2.1. Ideal Propagation Model

The TDE task for sound source detecting in a room can be formalized in several ways [8]. Each method is a compromise between the signal propagation model accuracy and the complexity of the mathematical description of the problem. The main acoustic signal propagation models are [8]: ideal propagation model, multipath propagation model, and reverberation model. In this work, we consider that the simulated microphones are equally capable of efficiently registering signals coming from any direction.

The ideal propagation model assumes that there is only one path from the signal source to each of the microphones. Let *s*_0_(*t*) be the signal emitted by the source. Then the signals of the receivers will be

(1) $$\begin{array}{l} s_{a}(t)=\alpha_{a}\cdot s_{0}(t-\tau_{a})+n_{a}(t),\\ s_{b}(t)=\alpha_{b}\cdot s_{0}(t-\tau_{b})+n_{b}(t). \end{array}$$

where *τ_a_*, *τ_b_* are lag values; *α_a_*, *α_b_* are signal attenuation coefficients; *n_A_*(*t*), *n_B_*(*t*) are random uncorrelated additive microphone noises. The values of *τ_a_*, *τ_b_* are determined by the geometric distances *r_a_*, *r_b_* from the signal source to the corresponding receiver

(2) $$\tau_{a}=\frac{r_{a}}{c},\;\tau_{b}=\frac{r_{b}}{c},$$

where *c* is the sound speed. Attenuation of signals *α_a_*, *α_b_* can be caused by various factors, however, in the simplest ideal case, exclusively source beam pattern and the scattering of the sound wave are considered and, so

(3) $$\alpha_{a}=\frac{k}{r_{a}{}^{2}},\;\alpha_{b}=\frac{k}{r_{b}{}^{2}},$$

where *k* is a constant coefficient.

In this case, the TDE is performed to get the value *τ_ab_* = *τ_b_* − *τ_a_* which is used further to determine the position of the sound source. Using the notations above and having redefined *t* = *t* − *τ_b_*, we can rewrite (1)

(4) $$\begin{array}{l} s_{a}(t)=\frac{k}{r_{a}{}^{2}}\cdot s_{0}(t+\frac{r_{b}-r_{a}}{c})+n_{a}(t),\\ s_{b}(t)=\frac{k}{r_{b}{}^{2}}\cdot s_{0}(t)+n_{b}(t). \end{array}$$

Expression (4) does not consider the influence of several physical factors, such as reflection and absorption of sound in a room.

Later, in the course of computational experiments with the ideal scenario, we will take that *k* = 1, since the target signal-to-noise ratio (SNR) can be achieved exclusively by changing the noise intensity.

### 2.2. Reverberation Model

The problem of the ideal propagation model is that the assumptions made do not correspond to the acoustic conditions of the real-world enclosed room. Firstly, there are always several paths for sound propagation between the source and the receiver due to the presence of reflected waves. Secondly, the absorption of sound energy by room surfaces has a significant effect on the recorded signal.

In accordance with the reverberation model, the received signals are described as follows

(5) $$\begin{array}{l} s_{a}(t)=\int_{0}^{T}h_{a}(\tau )\cdot s_{0}(t-\tau )\cdot d\tau \;+n_{a}(t),\\ s_{b}(t)=\int_{0}^{T}h_{b}(\tau )\cdot s_{0}(t-\tau )\cdot d\tau \;+n_{b}(t). \end{array}$$

where *h_a_* (*t*), *h_b_* (*t*) are room impulse response (RIR) functions. The complexity of application of (5) is in the practical difficulty of RIR determination. Acoustic measurements [18] or mathematical methods can be used to solve this problem. The image model method, first proposed in [19], is the most widespread among the latter. Alternatively, statistical methods [20] or methods based on geometric acoustics and ray tracing [21] can be used. To create realistic sound signals in this work, the image model method was used in the implementation of Lehman, Johansson and Nordholm [22,23].

### 2.3. Basic Phase Shift TDE

The phase TDE algorithm is based on obtaining information about the delay value from the cross-phase spectrum Φ*_ab_* of two signals. The algorithm for constructing the cross-phase spectrum is known from spectral analysis [14]. At the initial stage, the Fourier transforms *S_a_*(*f_k_*) and *S_b_*(*f_k_*) of the signals of each of the channels are determined

(6) $$S_{a}\left(f_{k}\right)=F_{D}\left(s_{a}(t_{i})\right),\;S_{b}\left(f_{k}\right)=F_{D}\left(s_{b}(t_{i})\right),$$

where *s_a_*(*t_i_*) and *s_b_*(*t_i_*) are series of *N* real samples of *s_a_*(*t*) and *s_b_*(*t*) signals sampled with an interval Δ; *F_D_* is the operator of short-time discrete Fourier transform (DFT); *S_a_*(*f_k_*) and *S_b_*(*f_k_*) are spectrums of the signals.

Further instantaneous cross-spectrum of signals $S_{ab}^{\left(q\right)}($*f_k_*) are calculated

(7) $$S_{ab}^{\left(q\right)}\left(f_{k}\right)=S_{a}^{\left(q\right)}{}^{*}\left(f_{k}\right)\times S_{b}^{\left(q\right)}\left(f_{k}\right),$$

where superscript (*q*) indicates the time instant *t_q_* = Δ∙*N*∙*q* of the beginning of the *q*-th time window; * is the element-wise complex conjugation*; ×* is the element-wise product. The final measurement of the cross-spectrum *S_ab_*(*f_k_*) is obtained by averaging the *Q* instantaneous spectrums

(8) $$S_{ab}^{}\left(f_{k}\right)=\frac{1}{Q}\sum_{q=0}^{Q-1}S_{ab}^{\left(q\right)}\left(f_{k}\right).$$

It should be noted that the application of (8) requires that the signal source remains stationary relatively to the receivers during the entire time of signal recording. If it is not, the spectral estimation *S_ab_*(*f_k_*) would not be correct. However, this assumption is normally relevant for the cross-spectrum. If we consider that neither source nor sensors are moving, the phase shift for each particular harmonic component will remain the same for all *Q* instantaneous spectrums. Therefore, coherent accumulation is applied this way to reduce the impact of the additive random noise.

To retrieve the set of phases, the phase cross-spectrum Φ*_ab_*(*f_k_*) is finally calculated

(9) $$\Phi_{ab}^{}\left(f_{k}\right)=U\left[\arg\left[S_{ab}^{}\left(f_{k}\right)\right]\right],$$

where U is an operator of phase unwrapping [24]; arg is the operator for defining the argument of a complex number.

All harmonic components presented in *s*_0_(*t*) will also be present in *s_a_*(*t*) and *s_b_*(*t*). In this case, the phase difference between the *k*-th harmonic components of *s_a_*(*t*) and *s_b_*(*t*) is determined by τ*_ab_*∙*f_k_*. Therefore, the estimation τ*_ab_* can be obtained as the coefficient of proportionality in the line equation of the approximating Φ*_ab_*(*f_k_*).

The value ${\hat{\tau }}_{ab}$ can be determined, for example, based on the criterion for minimizing the squared error function [14]. Let the error *e* be determined as

(10) $$e=\sum_{k}^{}{\left(\Phi_{ab}^{}\left(f_{k}\right)-\left({\overset{⌢}{\tau }}_{ab}\cdot 2\pi \cdot f_{k}+b_{ab}\right)\right)}^{2},$$

where $b_{ab}$ is a constant term. Then

(11) $$\left\{ \begin{array}{l} \frac{de}{d{\overset{⌢}{\tau }}_{ab}}=\;-2\cdot \sum_{k}^{}f_{k}\cdot \left(\Phi_{ab}^{}\left(f_{k}\right)-{\overset{⌢}{\tau }}_{ab}\cdot 2\pi \cdot f_{k}-b_{ab}\right),\\ \frac{de}{db_{ab}}=\;-2\sum_{k}^{}\left(\Phi_{ab}^{}\left(f_{k}\right)-{\overset{⌢}{\tau }}_{ab}\cdot 2\pi \cdot f_{k}-b_{ab}\right). \end{array}\right.$$

Equating the derivatives to zero in (11) results in

(12) $${\overset{⌢}{\tau }}_{ab}=\frac{\Delta \cdot N}{2\pi }\cdot \frac{D\cdot K-A\cdot C}{B\cdot K-A^{2}},$$

where values *A*, *C*, *B*, *D* can be computed with the proposed scheme

(13) $$A=\sum_{k}^{}k;\;B=\sum_{k}^{}k^{2};\;C=\sum_{k}^{}\Phi_{ab}^{}\left(f_{k}\right);\;D=\sum_{k}^{}k\cdot \Phi_{ab}^{}\left(f_{k}\right).$$

An advantage of the algorithm based on the use of (12) and (13) is that non-adjacent spectral bins can be used for TDE. It is optimal to choose k∈S, where *S* is a set of the most essential harmonic components of the signal *s*_0_(*t*).

### 2.4. Generalized Phase Spectrum TDE

A modification of the method described in the previous subsection can be used to localize stationary signal sources. The modified method was initially proposed in [15] and was named GPS TDE.

A distinctive feature of the generalized method is the use of real-valued frequency weight function *W*(*f_k_*) which is used to determine ${\hat{\tau }}_{ab}$. Similarly to (10), the weighted error in this case are introduced

(14) $$e=\sum_{k}^{}{\left[W\left(f_{k}\right)\cdot \left(\Phi_{ab}^{}\left(f_{k}\right)-\left({\overset{⌢}{\tau }}_{ab}\cdot 2\pi \cdot f_{k}+b_{ab}\right)\right)\right]}^{2}.$$

Obtaining a calculation formula for ${\hat{\tau }}_{ab}$ could be carried out in the same way as in the previous subsection

(15) $${\overset{⌢}{\tau }}_{ab}=\frac{\Delta \cdot N}{2\pi }\cdot \frac{\Lambda \cdot Κ-A\cdot \Theta }{Κ\cdot Β-A^{2}},$$

(16) $$Κ=\sum_{k}^{}W\left(f_{k}\right),A=\sum_{k}^{}k\cdot W\left(f_{k}\right),Β=\sum_{k}^{}k^{2}\cdot W\left(f_{k}\right),\;\Theta =\sum_{k}^{}\Phi_{ab}^{}\left(f_{k}\right)\cdot W\left(f_{k}\right),\Lambda =\sum_{k}^{}k\cdot \Phi_{ab}^{}\left(f_{k}\right)\cdot W\left(f_{k}\right).$$

It is clear from (14) that the functions *W*(*f_k_*) should be chosen in the way that its value is high if the useful signal prevails over noises at the *f_k_* frequency and differs little from zero in other cases. A set of five frequency weighting functions was investigated in [14]. Table 1 below shows the calculation formulas for these functions.

The coherence function γ^2^*_ab_* (*f_k_*) widely used for this purpose is calculated as

(17) $$\gamma^{2}{}_{ab}\;(f_{k})=\frac{{\left|\sum_{q=0}^{Q-1}\left(S_{a}{{}^{\left(q\right)}}^{*}\left(f_{k}\right)\cdot S_{b}{}^{\left(q\right)}\left(f_{k}\right)\right)\right|}^{2}}{\sum_{q=0}^{Q-1}{\left|S_{a}{}^{\left(q\right)}\right|}^{2}\cdot \sum_{q=0}^{Q-1}{\left|S_{b}{}^{\left(q\right)}\right|}^{2}}.$$

It should be noted that the computational scheme proposed in this section differs from the one in [14]. Equation (15) allows the unwrapped phase spectrum to not pass through the origin, as far as we used coefficient *b_ab_* in linear regression. This feature is practically important and will be addressed later. As far as *W*(*f_k_*) is based on spectral estimations, the generalized method should be applied carefully for signals that are non-stationary.

## 3. Results and Discussion

A series of computational experiments were carried out for a comparative evaluation of the algorithms. The human voice is commonly used for evaluation purposes in related studies [7,8]. Prior to the proposed study, we have tested algorithm performance for several speakers but did not find a significant difference in the results. Therefore, we have used the recording of one speaker and focused the study mainly on evaluating the impact of additive noise and multipath propagation in a reverberant environment.

A recording of a male speaker’s voice with additive random noise was used to produce a set of test signals. The noise-free sound was synthesized based on the recorded voice by each of two means: in accordance with (4) and in accordance with (5).

Additive noises were generated by software, then scaled and summed with the preprocessed recording. The spectral noise density was equal in the range from 0 to 1000 Hz. Signals and noises outside of this frequency range were not considered in the experiments. A similar approach to preparing the set of test signals was used in [25].

Noises of the same intensity were applied to both channels. At the same time, the intensity of the noise was set in such a way as to provide the target SNR relative to the root-mean-square value of the signals recorded by the sensors for the entire time of each instance of the experiment. When applying (1), the delay was introduced by shifting one copy of the record relative to another by an integer number of sampling intervals (*f_d_* = 44,100 Hz).

### 3.1. Experimental Setting

A set of stereo test records with a duration of about 20 s each were prepared for the study. The recording was analyzed in fragments of about 1 s during each instance of the experiment. At the same time, the analysis of each of the fragments was considered an independent experiment. The final estimations used to calculate the absolute error were obtained by averaging obtained values of the lag time.

The number of samples in each of the analyzed fragments was *L* = 40,960 (about 928.8 msec). The number of samples in the segment was taken to be *N* = 4096 (about 92.9 msec). Consequently, each piece of recording sound was fragmented into *Q* = 10 segments. When processing the results, the outputs corresponding to the segments of the recording, where pauses in speech predominated, were discarded.

**Figure 2:**
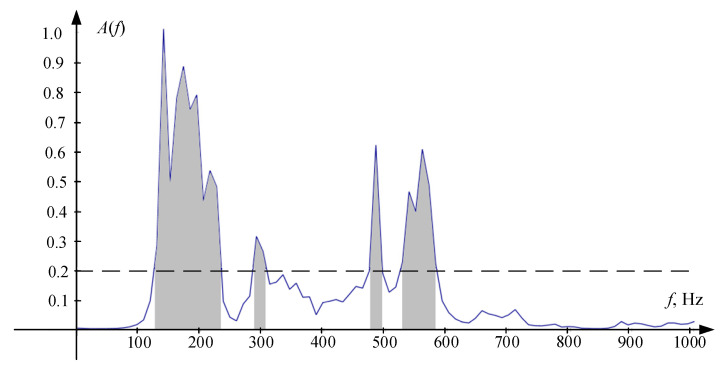
Raw signal power density spectrum. Frequency bins that are included in highlighted areas comprise the second set. Highlighted frequency bands are: 127–237 Hz, 285–305 Hz, 476–496 Hz, 531–580 Hz.

Two different sets of frequency bins were used when applying (16). The first set contained frequency bins corresponding to the condition *f_k_* ϵ [100 Hz, 850 Hz]. The second set contained four non-overlapping frequency bands shown below. The choice of such frequency intervals was carried out in accordance with the form of power density spectrum of the raw signal shown in Figure 2. The presented characteristic was obtained by averaging all instantaneous power density spectrums with a window of *N* = 4096 samples. The position of the cut-off level was chosen empirically to optimize the TDE operation in the absence of reverberations. It should be noted that the power density spectrum for different speakers or even for different speech fragments by this speaker would not remain the same. However, the proposed procedure will remain applicable regardless.

### 3.2. Simulation of the Small Room Environment

**Figure 3:**
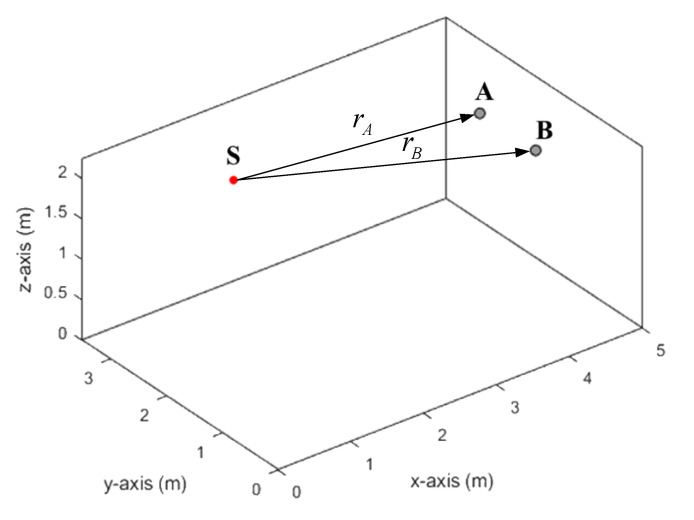
Source and microphones configuration in the model room. Source located in position S. Microphones are in positions A, B. Distances are *r_A_* = 3.041 m, *r_B_* = 3.354 m.

As noted above, creating a realistic sound signal in accordance with (5) requires obtaining RIR functions *h_a_* (*t*), *h_b_* (*t*). The MATLAB program prepared by Eric Lehman [22] was used to obtain these characteristics. When calculating the RIR, the room parameters and the configuration of the sensors were specified as shown in Figure 3. The dimensions of the room were 5 × 3.5 × 2.25 m. The source has coordinates (1.5, 2.75, 1.8), and the microphones (4.5, 1.25, 1.8) and (4.5, 2.25, 1.8).

**Figure 4:**
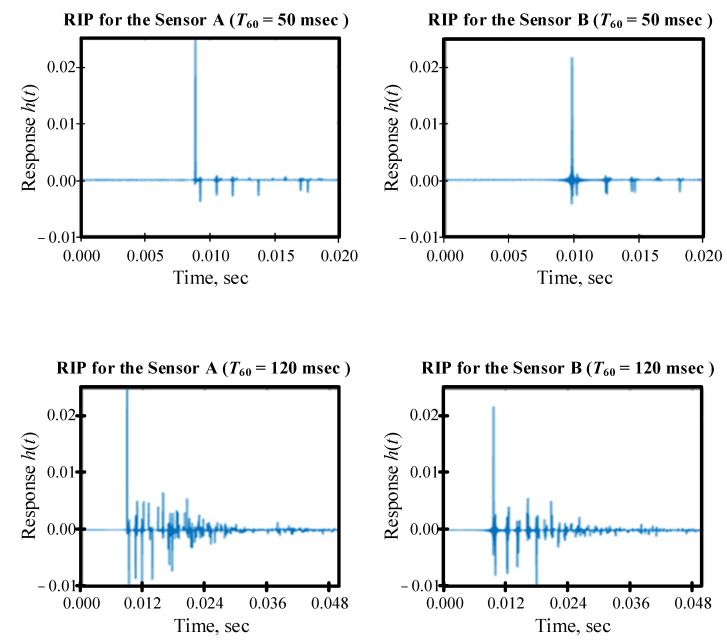
Room impulse responses for various reverberation times. True time delay is 0.923 msec.

The reverberation time (*T*_60_) was assumed to be 50 msec and 120 msec. The first value is compliant with the standards of a room intentionally designed for voice broadcasting. The second value is compliant with the requirements for verbal communication in an office space [26]. The synthesized RIRs are shown in Figure 4.

### 3.3. Comparison of GPS TDE Methods in Anechoic Environment

**Figure 5:**
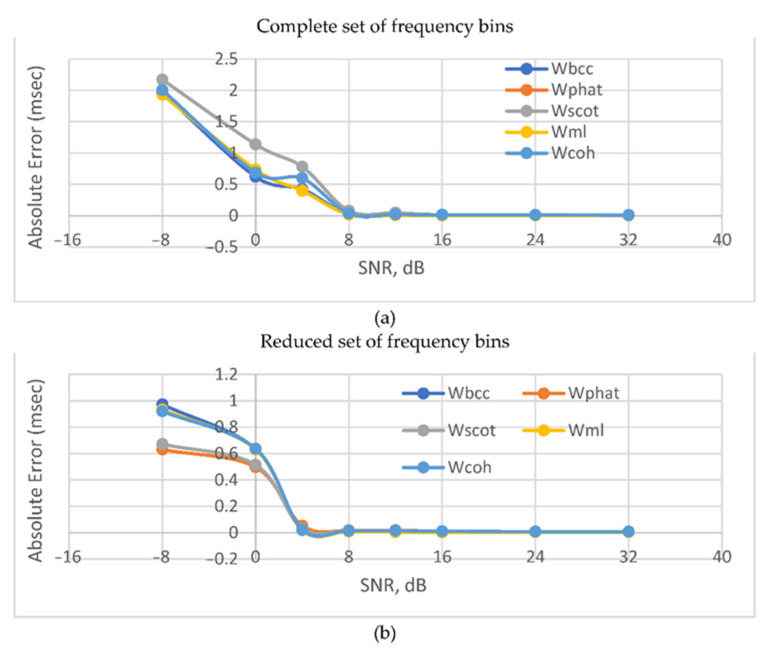
Absolute error vs SNR for anechoic room environment for: complete (**a**); and reduced (**b**) sets of frequency bins.

Table 2 shows the absolute TDE errors for various weight functions and the ideal signal propagation model. Figure 5 shows the dependence of TDE error on SNR.

Figure 5 shows that the use of a reduced number of frequency bins in (15) and (16) provides greater accuracy while increasing the intensity of in-band noises. At the same time, the use of the second reduced frequency set allows you to reduce the threshold SNR to 4 dB over which sharp drop in the accuracy manifests.

**Figure 6:**
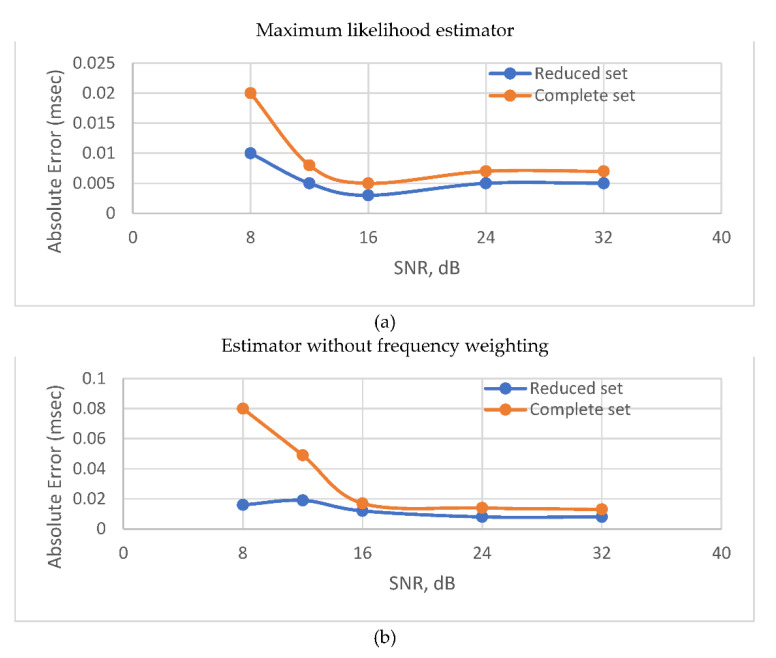
Absolute error vs SNR for anechoic room environment: (**a**) maximum likelihood weighting function (*W_ML_*); (**b**) no weighting was applied (*W_PHAT_*).

**Figure 7:**
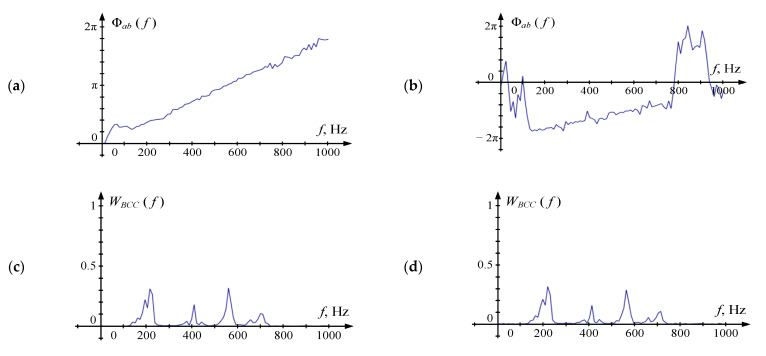

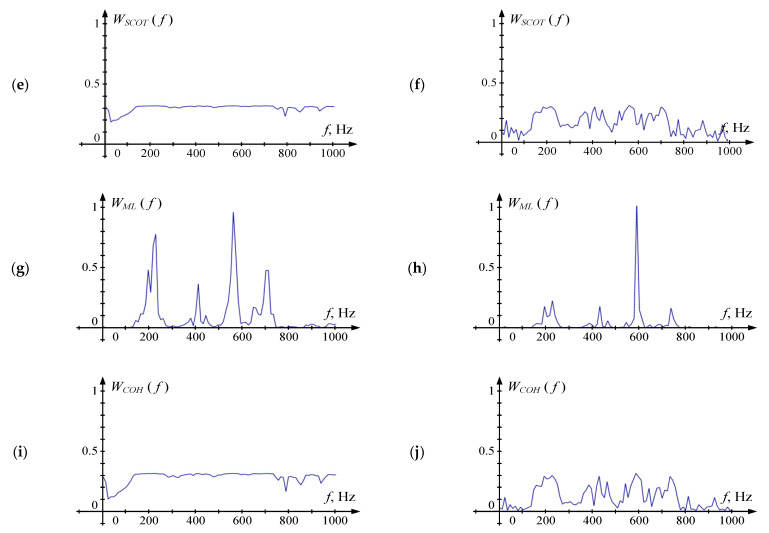
Sample phase cross spectrum Φ*_ab_* (*f_k_*) and weighting functions *W*(*f_k_*) for various SNR: (**a**,**b**) Φ*_ab_* (*f_k_*), (**c**,**d**) *W_BCC_*(*f_k_*), (**e**,**f**) *W_SCOT_*(*f_k_*), (**g**,**h**) *W_ML_*(*f_k_*), (**i**,**j**) *W_COH_*(*f_k_*). Figures (**a**,**c**,**e**,**g**,**i**) are obtained for SNR = 32 dB. Figures (**b**,**d**,**f**,**h**,**j**) are obtained for SNR = 4 dB. For *W_ML_* (*f_k_*) all values are normalized with the maximum value on the frequency band of interest.

Figure 6 shows the absolute TDE error for SNR ≥ 8 dB for *W_PHAT_* and *W_ML_*. When the noise intensity is not sufficient to go over the threshold, the estimators demonstrate the best possible performance in terms of accuracy regardless the noise level. When the SNR drops below the threshold level, the accuracy degrades gradually with the intensification of the noise. However, using a reduced set of frequency bins makes the contaminating effect of in-band noise less harsh. Notably, this is more obvious for *W_PHAT_* than for *W_ML_*. That can be explained by the fact that frequency weighting applied with ML estimator compensates for frequency bins where noise prevails over the signal. Despite the fact, that threshold SNR level appears in Figure 6 to be better for PHAT than for ML, the latter estimator surpasses the former in terms of accuracy in the single path scenario regardless of noise intensity. The frequency weighting function for the ML estimator is in Figure 7.

Figure 7 shows the form of Φ*_ab_* (*f_k_*) and all *W*(*f_k_*) in the absence of noise (SNR = 32 dB) and their presence (SNR = 4 dB). A part of the curve that is close to linear shape is clearly distinguished at Φ*_ab_*, in both cases, however, in the presence of noise, the corresponding frequency range is significantly narrower. It should be noted that Φ*_ab_* in the absence of noise passes through the origin and behaves as described in [14]. However, when the signal is contaminated with the noise, Φ*_ab_* is offset relative to the abscissa axis. This can be explained by the fact that there is no voice signal on frequencies up to 100 Hz, so the prevalence of the noise in this band results in an unpredictable offset of the unwrapped phase spectrum. That makes the estimation technique proposed in [14] not relevant for this task.

The shape of *W_SCOT_* and *W_COH_* is close to a line parallel to the time axis in the absence of noise. In the presence of noise, a high level of *W_SCOT_* and *W_COH_* is observed in the intervals where the cross-power spectrum |*S_ab_*| has high values. *W_BCC_* form follows the shape of |*S_ab_*| and does not differ significantly in the presence of noise and their absence. Four areas of high values are visible at the *W_ML_* corresponding to the Φ*_ab_* regions that are best approximated by the line.

### 3.4. Comparison of GPS TDE Methods in Reverberant Environment

Table 3 and Table 4 summarize the average TDE absolute errors for different weighting functions, reverberation model and different reverberation times.

**Figure 8:**
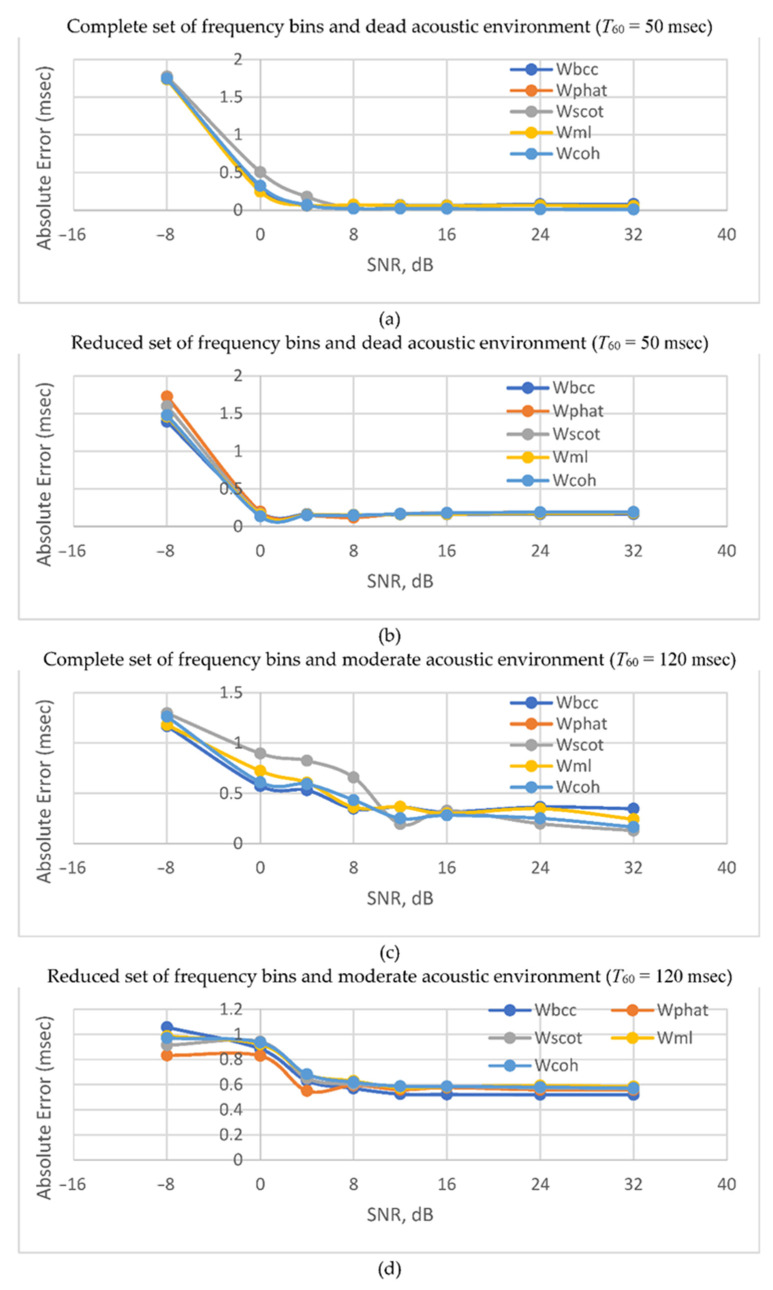
Absolute error vs SNR for reverberant room environment. For subfigures (**a**,**b**) *T*_60_ = 50 msec. For figures (**c**,**d**) *T*_60_ = 120 msec. Reduced set was used for (**b**,**d**). Complete set was used for (**a**,**c**).

Figure 8 shows that in the presence of reflected signals, the ML estimator is inferior in accuracy to the SCOT and COH estimators, especially in the absence of additive noises. At the same time, the accuracy turns out to be significantly lower than in the previous case. This can be explained by the correlation of the signals with their reflected copies. In the presence of reverberations and intense noises, none of the functions show any accuracy advantage. The latter makes it useful to apply the BPS TDE method (PHAT) as the simplest one.

The use of the second set of frequency bins provides an advantage in accuracy only in conditions of noise dominance (SNR ≤ 0 dB). The use of the complete set of frequency bins provides significantly better accuracy in other cases.

Figure 8 shows the dependence of TDE error on SNR graphically.

**Figure 9:**
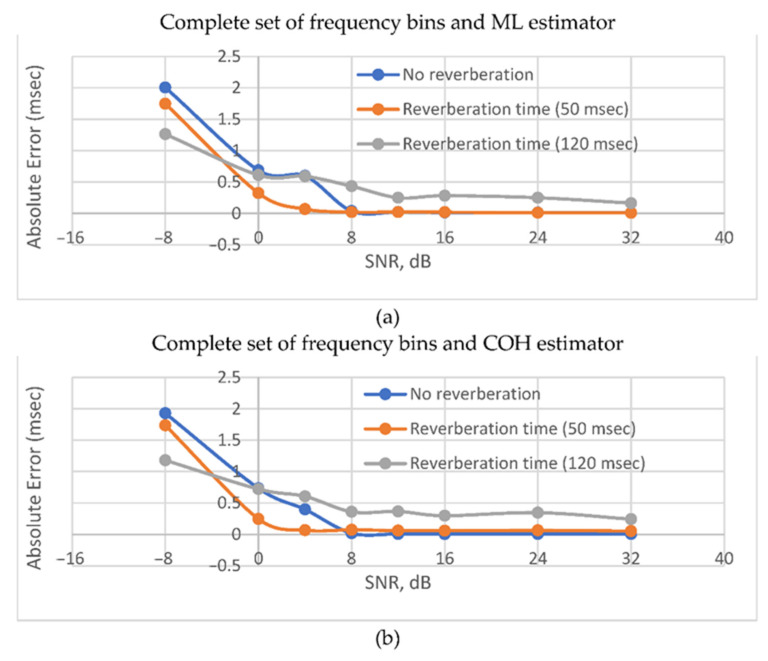
Absolute error vs SNR for various reverberation times and the complete set of frequency bins: (**a**) *W_ML_*; and (**b**) *W_COH_* frequency weighting functions were applied.

Figure 9 shows the results of using GPS TDE for various acoustic conditions of the environment. It is clear from the figure that the reverberation time increase leads to a drastic increase in the error both in the presence and absence of noise. However, with the dominance of noise over the signal, the presence of reflected copies has a positive effect on accuracy. However, even if this is the case, the TDE error remains unacceptably high for a significant part of practical applications.

**Figure 10:**
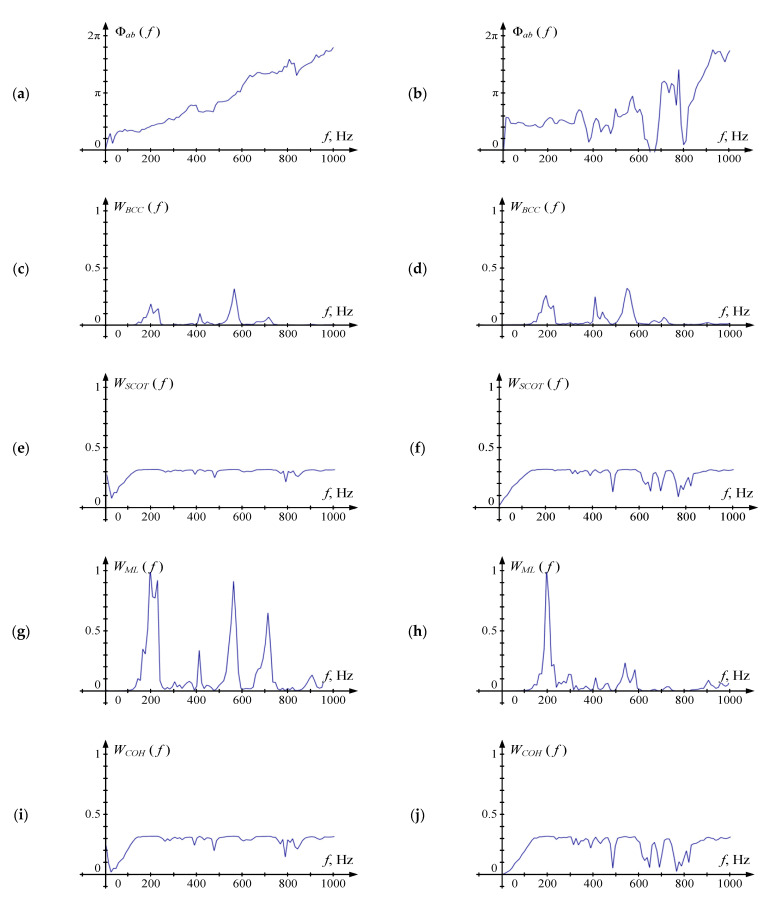
Sample phase cross spectrum Φ*_ab_* (*f_k_*) and weighting functions *W*(*f_k_*) for various reverberation times: (**a**,**b**) Φ*_ab_* (*f_k_*), (**c**,**d**) *W_BCC_*(*f_k_*), (**e**,**f**) *W_SCOT_*(*f_k_*), (**g**,**h**) *W_ML_*(*f_k_*), (**i**,**j**) *W_COH_*(*f_k_*). Figures (**a**,**c**,**e**,**g**,**i**) are obtained for *T*_60_ = 50 msec. Figures (**b**,**d**,**f**,**h**,**j**) are obtained for *T*_60_ = 120 msec. For *W_ML_* (*f_k_*) all values are normalized with the maximum value on the frequency band of interest.

Figure 10 shows the form of Φ*_ab_* (*f_k_*) and all *W*(*f_k_*) for different values of reverberation time (*T*_60_). All graphics in Figure 7 and Figure 10 are obtained for one and the same fragment of the original signal. It can be seen from the form of Φ*_ab_* that an increase in the reverberation time leads to a distortion of the frequency response form and a decrease in the estimate accuracy. At the same time, the distortions observed for *W_SCOT_* and *W_COH_* are not as significant as they were in the absence of reverberations and the presence of noises. This can be explained by the fact that the reflected signals are mutually correlated, and their presence does not contribute to a significant decrease in the level of signal coherence. The correlation of the reflected signals also affects at the shape |*S_ab_*| and, therefore, at the *W_BCC_* form. The *W_ML_* form also changes significantly with an increase in the reverberation time, while the regions of high values also correspond to the linear sections Φ*_ab_*. At *T*_60_ = 120 msec, the number of such sections becomes smaller which negatively affects the accuracy.

## 4. Conclusions

This study investigated GPS TDE in relation to the problem of localizing a sound source in a small room. The suggested TDE algorithm is based on the analysis of the phase response form which makes it possible to estimate the time by analyzing an arbitrary set of spectral bins.

To assess the algorithm’s applicability and efficiency, a series of computational experiments were performed to simulate the speaker positioning within a small room. To simulate room acoustics, the image model implemented by Lehman and Johanson [23] was used. During the course of the experiment, the SNR at the signal receivers was varied, as well as the room reverberation time.

The fundamental applicability of the suggested algorithm was shown due to the performed experiment. In the absence of noises and echo, GPS TDE demonstrates an accuracy comparable to the sampling error at *f_d_* = 44,100 Hz (about 0.01 s). A decrease in accuracy is expected in the absence of echo but at an increase in the intensity of additive noise. However, narrowing of the frequency range over which TDE is performed helps to maintain accuracy under moderate noises (SNR > 4 dB). The best accuracy characteristics are provided by the ML GPS estimator.

When an echo occurs, TDE accuracy downgrades significantly. The reflected signals are correlated, and, therefore, introduce extra noise to the correlogram. In this case, the use of a reduced set of spectral bins affects the accuracy negatively. Even with insignificant reverberations, corresponding to an acoustical very dead room and the absence of noises, the ML GPS estimator demonstrates a relatively low accuracy. The SCOT and COH GPS estimators show the best results. In conditions of higher reverberations, the TDE error increases significantly in comparison to the ideal case and makes the use of the GPS method ineffective. In practice, however, the influence of echo can be lower, as real-world microphones are not omnidirectional.

Even though the suggested method is inferior to analogs in a few aspects, its advantage remains high computational efficiency. The suggested computational scheme, when using a relatively small number of adjacent frequency samples for TDE, allows the use of Goertzel’s algorithm instead of FFT [27]. This is essential for embedded computers with memory constraints. Additionally, the use of well-known implementations of the Goertzel algorithm designed for phase detection [28] will make it possible to re-evaluate the spectral characteristics of the signal with new data arrival. The latter is useful for solving the problem of positioning a mobile acoustic source. Further studies will be devoted to the testing of this hypothesis.

## Figures and Tables

**Table 1:** Weight functions.

Method	Nomenclature	Formula
BCC	*W_BCC_* (*f_k_*)	|*S_ab_* (*f_k_*)|/max(|*S_ab_* (*f_k_*)|)
PHAT	*W_PHAT_* (*f_k_*)	1
SCOT	*W_SCOT_* (*f_k_*)	γ*_ab_* (*f_k_*)
ML	*W_ML_* (*f_k_*)	γ^2^*_ab_* (*f_k_*)/[1 − γ^2^*_ab_* (*f_k_*)]
COH	*W_COH_* (*f_k_*)	γ^2^*_ab_* (*f_k_*)

**Table 2:** Absolute error of GPS TDE with ideal propagation model.

Set	SNR	Mean Absolute Error (msec)				
	(dB)	*W_BCC_* (*f_k_*)	*W_PHAT_* (*f_k_*)	*W_SCOT_* (*f_k_*)	*W_ML_* (*f_k_*)	*W_COH_* (*f_k_*)
First	32	0.008	0.013	0.012	0.007	0.011
	24	0.007	0.014	0.016	0.007	0.013
	16	0.005	0.017	0.020	0.005	0.016
	12	0.008	0.049	0.031	0.008	0.023
	8	0.020	0.080	0.053	0.020	0.038
	4	0.425	0.782	0.626	0.399	0.600
	0	0.623	1.139	0.893	0.735	0.687
	−8	1.961	2.171	2.100	1.931	2.006
Second	32	0.005	0.008	0.009	0.005	0.009
	24	0.005	0.008	0.009	0.005	0.009
	16	0.004	0.012	0.011	0.003	0.012
	12	0.008	0.019	0.017	0.005	0.015
	8	0.011	0.016	0.018	0.010	0.015
	4	0.020	0.053	0.030	0.022	0.024
	0	0.634	0.497	0.514	0.631	0.637
	−8	0.973	0.631	0.672	0.934	0.921

**Table 3:** Absolute error of GPS TDE with reverberation model (*T*_60_ = 50 msec).

Set	SNR	Mean Absolute Error (msec)				
	(dB)	*W_BCC_* (*f_k_*)	*W_PHAT_* (*f_k_*)	*W_SCOT_* (*f_k_*)	*W_ML_* (*f_k_*)	*W_COH_* (*f_k_*)
First	32	0.081	0.012	0.009	0.054	0.009
	24	0.080	0.014	0.010	0.065	0.012
	16	0.063	0.019	0.017	0.059	0.019
	12	0.066	0.021	0.015	0.061	0.021
	8	0.072	0.027	0.017	0.073	0.022
	4	0.062	0.177	0.097	0.067	0.069
	0	0.272	0.505	0.407	0.247	0.324
	−8	1.735	1.775	1.746	1.736	1.747
Second	32	0.166	0.195	0.195	0.182	0.194
	24	0.165	0.190	0.192	0.178	0.192
	16	0.163	0.181	0.183	0.169	0.181
	12	0.161	0.171	0.171	0.163	0.168
	8	0.155	0.122	0.148	0.153	0.150
	4	0.165	0.151	0.156	0.157	0.150
	0	0.190	0.199	0.168	0.162	0.137
	−8	1.392	1.727	1.598	1.460	1.478

**Table 4:** Absolute error of GPS TDE with reverberation model (*T*_60_ = 120 msec).

Set	SNR	Mean Absolute Error (msec)				
	(dB)	*W_BCC_* (*f_k_*)	*W_PHAT_* (*f_k_*)	*W_SCOT_* (*f_k_*)	*W_ML_* (*f_k_*)	*W_COH_* (*f_k_*)
First	32	0.346	0.129	0.146	0.243	0.164
	24	0.364	0.198	0.208	0.347	0.251
	16	0.315	0.327	0.320	0.299	0.282
	12	0.365	0.194	0.212	0.366	0.251
	8	0.348	0.658	0.578	0.361	0.433
	4	0.531	0.825	0.768	0.606	0.592
	0	0.572	0.897	0.842	0.723	0.611
	−8	1.169	1.297	1.291	1.181	1.263
Second	32	0.519	0.558	0.567	0.584	0.571
	24	0.520	0.559	0.574	0.592	0.580
	16	0.522	0.577	0.586	0.583	0.586
	12	0.525	0.560	0.586	0.574	0.587
	8	0.570	0.594	0.604	0.629	0.619
	4	0.631	0.550	0.651	0.682	0.683
	0	0.884	0.829	0.942	0.921	0.935
	−8	1.057	0.832	0.914	0.985	0.971

## Data Availability

For the experiments, a model of a room’s acoustic environment was used to synthetize test data. The model is implemented by Eric Lehman as MATLAB program and can be downloaded here http://www.eric-lehmann.com/ (last accessed on 17 November 2021).
